# Validation of maternal reported pregnancy and birth characteristics against the Medical Birth Registry of Norway

**DOI:** 10.1371/journal.pone.0181794

**Published:** 2017-08-04

**Authors:** Svein Magne Skulstad, Jannicke Igland, Ane Johannessen, Randi Jacobsen Bertelsen, Marianne Lønnebotn, Ernst Reidar Omenaas, Cecilie Svanes, Francisco Gomez Real

**Affiliations:** 1 Dept. of Clinical Science, University of Bergen, Bergen, Norway; 2 Dept. of Immunology and Transfusion Medicine, Haukeland University Hospital, Bergen, Norway; 3 Department of Global Public Health and Primary Care, University of Bergen, Bergen, Norway; 4 Centre for International Health, University of Bergen, Bergen, Norway; 5 Dept. of Occupational Medicine, Haukeland University Hospital, Bergen, Norway; 6 Centre for Clinical Research, Haukeland University Hospital, Bergen, Norway; 7 Dept. of Obstetrics and Gynecology, Haukeland, University Hospital, Bergen, Norway; Univesity of Iowa, UNITED STATES

## Abstract

Studies using mothers’ self-reported information on birth and pregnancy characteristics are common, but the validity of such data is uncertain. We evaluated questionnaire data from the RHINE III study on reproductive health provided by 715 mothers from Bergen, Norway, about their 1629 births between 1967 and 2010, using the Medical Birth Registry of Norway (MBRN) as gold standard. Validity of dichotomous variables (gender, preterm birth [<37 weeks’ gestation], postterm birth [>42 weeks’ gestation], induction of labour, forceps delivery, vacuum delivery, caesarean section, were assessed by sensitivity, specificity, positive and negative predictive values (PPV and NPV) and Cohen’s kappa. Paired t-test, Pearson’s correlation coefficient and Bland-Altman plots were used to validate birthweight, stratified by mother’s level of education, parity, birth year and child’s asthma status. Child’s gender and caesarean section showed high degree of validity (kappa = 0.99, sensitivity and specificity 100%). Instrumental delivery and extremely preterm birth showed good agreement with sensitivity 75–92%. Preterm birth and induction of labour showed moderate agreement. Post-term delivery was poorly reported. The validity appeared to be independent of recall time over 45 years, and of the child’s asthma status. Maternally reported birth and pregnancy information is feasible and cheap, showed high validity for important birth and pregnancy parameters, and showed similar risk-associations compared to registry data.

## Introduction

Over the past decades, there has been increasing interest in the effects of prenatal exposures on disease predisposition in adulthood [1, 2]. In many of these studies, birthweight, pregnancy characteristics and birth complications are used as markers of the prenatal milieu. Data on measured birth and pregnancy characteristics are often not available, particularly in studies of adults, and researchers thus have to base their analyses on information recalled by the mother.

There are studies validating maternally reported data against nation-wide registry data [3], while other studies validate against medical hospital records [4–7]. Maternal remembrance has been found to be very good for birthweight [3–8], good for gestational age at delivery [3, 5, 9], and satisfactory for mode of delivery [6, 9]. However, the effect of time since birth on the quality of the report have not been investigated in any of these studies. A potential effect of recall time is of importance as these characteristics are often recorded when the child is adult and chronic diseases are already present. Some evidence exist that the importance of a past event influences the recall of that event many years later [10]. Another potential source of bias related to maternally reported data, is the possibility that later disease status in the child might influence maternal recall of birth characteristics and pregnancy complications if this is reported after onset of disease in the offspring. Mothers of children with a disease might be more susceptible to report negative pregnancy and birth characteristics than mothers of healthy children, this could contribute to observe stronger associations of early life risks with subsequent disease.

The aims of the present study were to investigate 1) the validatity of maternally recorded birthweight, gestational age, mode of delivery (vaginal delivery/caesarean section) use of forceps/vacuum and induction of labour; 2) whether the validity of mothers’ reported birthweight as well as other maternal characteristics differed by the number of years from birth until report. We used a datasource with information about asthma-status in the child, known at the time the mother reported information about birth and pregnancy, and thus used this as an example to investigate whether mothers’ reporting differed if the child had a specific disease (asthma) or not.

## Methods

The Respiratory Health In Northern Europe (RHINE) study was initially recruited as part of the European Community Respiratory Health Survey (ECRHS) I stage I (www.ecrhs.org). The RHINE III constitutes the second questionnaire follow-up of a population-based cohort from seven Northern European centres (Bergen in Norway; Umeå, Gothenburg and Uppsala in Sweden; Aarhus in Denmark; Reykjavik in Iceland and Tartu in Estonia)(www.rhine.no) [11]. An extensive women’s health questionnaire was sent to female responders of the last follow-up, providing details about reproductive health. The study population in the present analysis included women from the study centre in Bergen, Norway.

**Fig 1:**
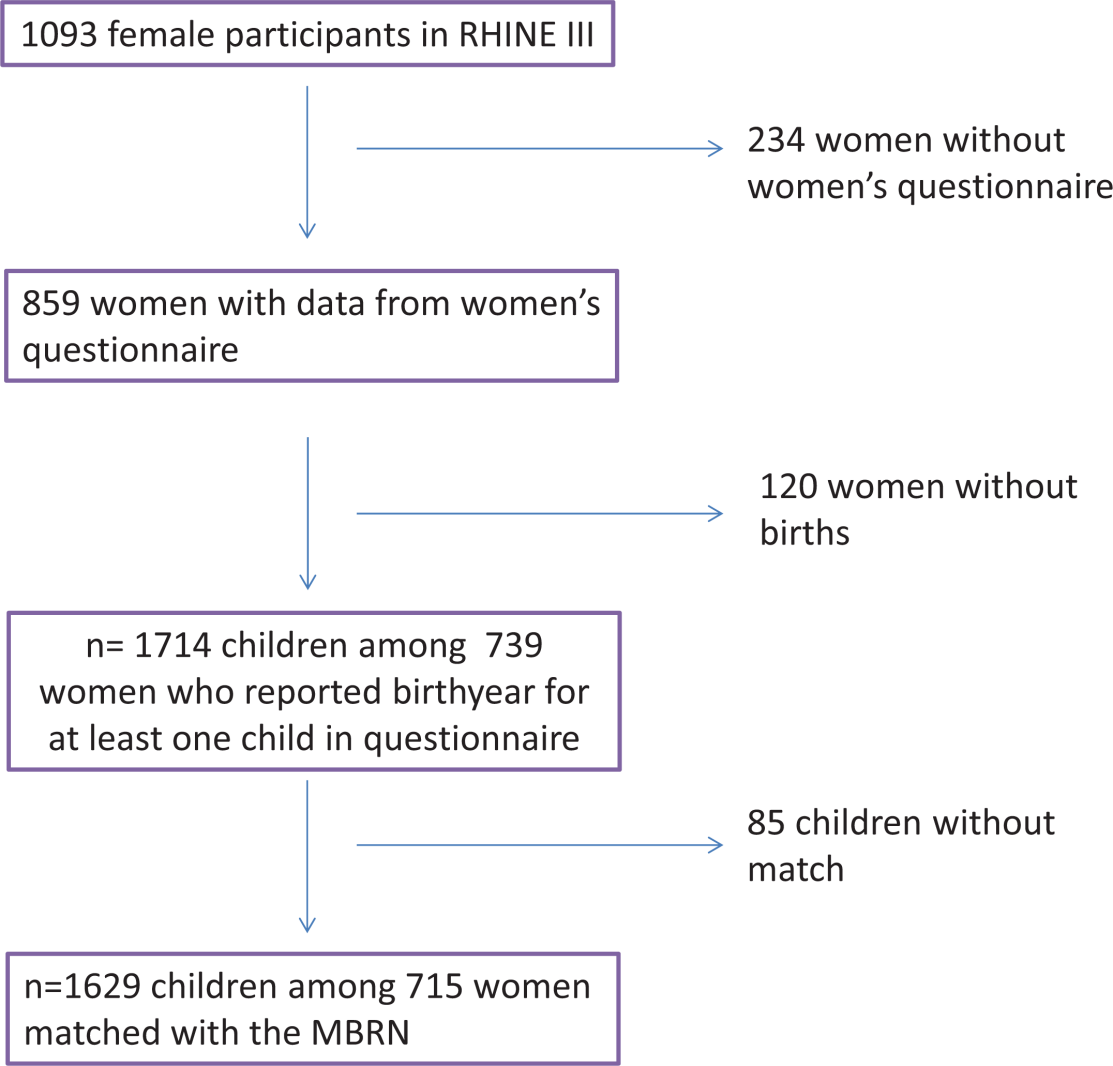
Flowchart for the definition of the study population of mothers in RHINE III.

In the third stage of RHINE in Bergen between 2010 and 2012, 2364 individuals participated, among which 1093 were women. Out of these, 859 women filled in and returned the women’ s health questionnaire (Fig 1). All women who had given birth were asked to fill in a set of questions regarding pregnancy and child birth for up to six children, including stillbirths. For each child they were asked to report year of birth, the child’s gender, birthweight in kilograms with one decimal, gestational age at the time of birth in four categories: Extremely preterm (before 32 weeks), preterm (32–36 week), term (37–42 weeks) and postterm (>42 weeks), complications during pregnancy and childbirth, whether the labour was induced or not and mode of delivery (spontaneous vaginal, forceps, vacuum or caesarean section). The dichotomous variable low birth weight was defined according to birthweigt under or above 2.5 kg, in accordance to WHO definitions [12]. A total of 739 women reported birth year for at least one child. The total number of children reported was 1714.

The Medical Birth Registry of Norway (MBRN) is a nation-wide population-based registry established in 1967. Registration of information on all live births and stillbirths in Norway from 16 weeks of gestation is mandatory. Data from the antenatal forms carried by all pregnant women are transferred to the MBRN registration form after delivery, together with data recorded at the maternity ward by the midwives. The variables in the registry have been validated against patient records and found to be satisfactory [13–17]. In one study, the diagnosis of pre-eclampsia was confirmed in 63.6% (1967–2005) based on the medical records. PPV was 82.0% [13]. Another study validated the registration of diseases in women in the MBRN using prescribed medicines and reimbursement codes registered in the Norwegian Prescription Database (NorPD) as the ‘gold standard.’ A sensitivity of the registration of type 1 diabetes of 90% was observed, while PPV was 56% and specificity 100%. The sensitivity for epilepsy was 74% (PPV 37%, specificity 100%). The sensitivity of the registration of asthma was 51% (PPV 46%, specificity 98%), with higher sensitivity for those with more serious disease [15].

Based on the RHINE III mothers’ unique personal identification numbers and self-reported birth years for the children we were able to match 1629 children (95%) from 715 RHINE III mothers with data from the MBRN (Fig 1). Maternally reported information about the following variables recorded in RHINE III were validated against corresponding information in the MBRN: gender, birthweight, preterm birth (<37 weeks’ gestation), postterm birth (>42 weeks gestation), induction of labour, forceps delivery, vacuum delivery and caesarean section.

### Statistical methods

Descriptive statistics about the mothers and the children were reported as mean (SD) for numerical variables, median and inter-quartile range for discrete variables and counts and percentages for categorical variables.

Validity of dichotomous variables was assessed by sensitivity, specificity, positive and negative predictive values (PPV and NPV), and Cohen’s kappa. Data from the MBRN were treated as the gold standard in all analyses. Mother’s self-report of birthweight for her child was validated using paired Pearson’s correlation coefficient and constant-only linear regression models with the difference between mother’s reported birthweight and the birthweight in the MBRN as the dependent variable. The estimated constant with confidence intervals indicates systematic bias. Confidence intervals were corrected for clustering within mothers by using a clustered sandwich estimator for the standard errors. We stratified the analyses by level of education, the number of children reported by the mother, calendar year of birth and asthma status in the child at age 10. To investigate systematically deviations between self-reported birthweight and birthweight in MBRN we made graphs of paired differences against the number of recall years and a Bland-Altman plot [18], which plots the paired difference between self-reported birthweight and birthweight in the MBRN against the mean of the paired birthweights. Limits of agreement were corrected for repeated reported birthweights within mothers.

STATA (StataCorp, College Station, TX, USA), version IC 14.0 was used in all analyses.

## Results

The study population comprised 1629 children of 715 mothers successfully matched with the MBRN (Fig 1). There were on average 2.3 children per mother and mother’s mean age was 51.4 (SD: 6.9) years when she filled in the questionnaire (Table 1). The children were born between 1967 and 2010 and the mean number of recall years for the mother, and hence also the age of the child at the time of reporting, was 22.7 (SD: 9.2) years. Fifty percent of the children were boys and mean birthweight was 3.5 (SD: 0.6) kg. Approximately 15% of the births were reported as postterm (> 42 weeks’ gestation) and 8% were reported as preterm (< 37 weeks’ gestation). The birth was reported to be induced for 19% of the children and the use of forceps, vacuum and caesarean section was reported for 5.1%, 2.3% and 9.3% of the births, respectively.

**Table 1:** Characteristics of mothers, and characteristics of pregnancies and births as reported by the mothers in the RHINE III questionnaire.

**Mother’s characteristics**		
N Mothers		715
N Children per mother, median (IQR*)		2 (2–3)
Mother’s age when filling out questionnaire, mean (SD)		51.4 (6.9)
**Mother’s marital status when filling out questionnaire, n (%)**		
	Married/Cohabitant	594 (83.1)
	Widowed/Divorced/Separated	84 (11.8)
	Single	26 (3.6)
	Do not wish to answer	6 (0.8)
Mother’s education**, n(%)		
	Primary	55 (7.7)
	Secondary	292 (40.8)
	University/College	363 (50.8)
**Mother’s reported characteristics of pregnancies/births**		
N Children		1629
Birthyear of children, min-max		1967–2010
Recall years, mean (SD)		22.7 (9.2)
Gender of children, n(%)		
	Boys	814 (50.0)
	Girls	752 (46.2)
	Missing	63 (3.9)
Birthweight in kg, mean (sd)		3.5 (0.6)
Low birthweight (<2.5 kg), n(%)		
	No	1392 (85.5)
	Yes	73 (4.5)
	Missing	164 (10.1)
Gestational age at birth		
	<32 weeks	29 (1.8)
	32–36 weeks	101 (6.2)
	37–42 weeks	1220 (74.9)
	>42 weeks	253 (15.5)
	Missing	26 (1.6)
Induction of labour, n(%)		312 (19.2)
Mode of delivery		
	Spontaneous vaginal	1260 (77.4)
	Forceps	83 (5.1)
	Vacuum	37 (2.3)
	Caesarean section	152 (9.3)
	Missing	97 (6.0)

*IQR:Inter quartile range

**According to the question: “Please mark the educational level which best describes your level: 1) Primary school 2) Lower or upper secondary 3) College or university

Validated against the Medical Birth Registry of Norway (Table 2), gender of the child and caesarean section were highly correctly reported by the mother with a kappa of 0.99 and a sensitivity and specificity of almost 100%. Use of forceps and vacuum, low birthweight and extremely preterm birth showed good agreement with MBRN with kappa values ranging from 0.73 to 0.85 and sensitivity between 75% and 92%. Preterm birth and induction of labour showed moderate agreement, specificity above 90% but sensitivity 73% - 81%, PPV 46% - 56%, and NPV 94% - 99%. Post-term delivery was poorly reported with a kappa of 0.38 and sensitivity of only 50%.

**Table 2:** Validity of dichotomous pregnancy and birth- characteristics reported by the mothers in RHINE III, with information in the MBRN used as the gold standard.

		Prevalence in questionnaire*	Sensitivity (95% CI)	Specificity (95% CI)	PPV (95% CI)	NPV (95% CI)	Kappa
**Boys**		813/1564	99.5 (98.7–99.9)	99.6 (98.8–99.9)	99.6 (98.6–99.9)	99.5 (98.6–99.9)	0.99
**Preterm delivery**		122/1516	73.0 (72.6–81.9)	96.0 (94.9–97.0)	53.3 (44.0–62.4)	98.3 (97.4–98.9)	0.60
**Preterm delivery by asthma status in the child**							
	No asthma	90/1219	71.9 (59.2–82.4)	96.5 (95.4–97.5)	51.7 (40.8–62.4)	98.5 (97.7–99.1)	0.57
	Asthma	17/114	72.7 (39.0–94.0)	91.3 (83.2–95.3)	44.4 (21.5–69.2)	96.9 (91.3–99.4)	0.51
**Extremely preterm delivery (<32 weeks)**		25/1516	75.0 (53.3–90.2)	99.5 (99.0–99.8)	72.0 (50.6–87.9)	99.6 (99.1–99.9)	0.73
**Postterm delivery (>42 weeks)**		237/1516	50.2 (43.2–57.3)	89.8 (88.0–91.3)	43.5 (37.1–50.0)	92.0 (90.4–93.5)	0.38
**Induction of labour**		312/1620	70.1 (64.0–75.7)	90.1 (88.4–91.6)	56.4 (50.7–62.0)	94.3 (92.9–95.5)	0.55
**Use of forceps**		83/1532	82.4 (71.8–90.3)	98.5 (97.7–99.1)	73.5 (62.7–82.6)	99.1 (98.5–99.5)	0.77
**Use of vacuum**		37/1532	75.6 (60.5–87.1)	99.8 (99.4–100)	91.9 (78.1–98.3)	99.3 (98.7–99.6)	0.82
**Caesarean section**		152/1532	100 (97.6–100)	99.9 (99.5–100)	98.7 (95.3–99.8)	100 (99.7–100)	0.99

*Denominators vary between the different characteristics due to missing values in the questionnaire data and in the MBRN. Only births with data in both the questionnaire and in the MBRN are included for each characteristic

*Sensitivity*: The proportion with the characteristic correctly identified

*Specificity*: The proportion without the characteristic correctly identified

*PPV*: Positive Predictive Value

*NPV*: Negative Predictive Value

When stratifying by asthma in the child as reported by the mother, no differences in validity of the maternally reported birth characteristics could be identified (Table 2), nor did stratification by recall years (S1 Table).

Among the 1464 children with valid information on birthweight in both the questionnaire and the MBRN the correlation between mother’s reported birthweight and birthweight in the MBRN was 0.95 (Table 3). The mean (SD) of the paired difference between mother’s reported birthweight and the birthweight registered in the MBRN was -0.03 (0.20) kg, indicating a slightly lower reported birthweight in the questionnaire (p<0.05). Stratification by education, number of children reported by the mother, birth year of the child and asthma status of the child showed very little variation in the correlation coefficient and the paired differences with no significant differences between subgroups. The mean difference was in general small (0.04 kg or less) but statistically significant for several subgroups with large sample size.

**Table 3:** The validity of mother’s recall of birth weight in terms of Pearson correlation coefficients and paired t-test with stratification on mother’s level of education, number of children reported by the mother and birth cohort.

		**n**	**Corr**	**Birthweight in questionnaire in kg, mean (SD)**	**Birthweight in MBR in kg,** **mean (SD)**	**Paired difference in kg,** mean (95% CI)*
Total		1464	0.95	3.46 (0.62)	3.49 (0.60)	-0.03 (0.04, -0.01)*
By level of education						
	Primary	107	0.95	3.50 (0.55)	3.46 (0.55)	0.03 (0.01, 0.08)
	Secondary	552	0.94	3.45 (0.62)	3.48 (0.61)	-0.03 (-0.05, -0.01)
	University/College	793	0.98	3.47 (0.61)	3.51 (0.60)	-0.03 (-0.05, -0.02)
By total number of children						
	1 child	98	0.93	3.45 (0.58)	3.46 (0.57)	-0.01 (-0.05, 0.03)
	2–6 children	1366	0.95	3.46 (0.62)	3.49 (0.61)	-0.03 (-0.04, -0.01)
By number of recall years						
	≤20 years	643	0.96	3.52 (0.58)	3.56 (0.57)	-0.03 (-0.05, -0.02)
	>20 years	643	0.93	3.41 (0.64)	3.43 (0.62)	-0.02 (-0.04, -0.004)
By birth cohort						
	1967–1979	270	0.90	3.43 (0.59)	3.43 (0.54)	0.002 (-0.03, 0.04))
	1980–1989	482	0.96	3.41 (0.67)	3.45 (0.65)	-0.04 (-0.06, -0.02))
	1990–1999	541	0.94	3.51 (0.57)	3.54 (0.57)	-0.03 (-0.05, -0.01)
	2000–2010	171	0.97	3.50 (0.65)	3.53 (0.64)	-0.03 (-0.05, -0.001))
By asthma status in the child						
	No asthma before age 10	1271	0.94	3.47 (0.59)	3.50 (0.58)	-0.03 (-0.04, -0.01)
	Asthma before age 10	82	0.92	3.48 (0.83)	3.51 (0.68)	-0.03 (-0.09, 0.02)

^*^Estimated using a constant only linear regression model with robust standard errors to account for clustering within mothers

**Fig 2:**
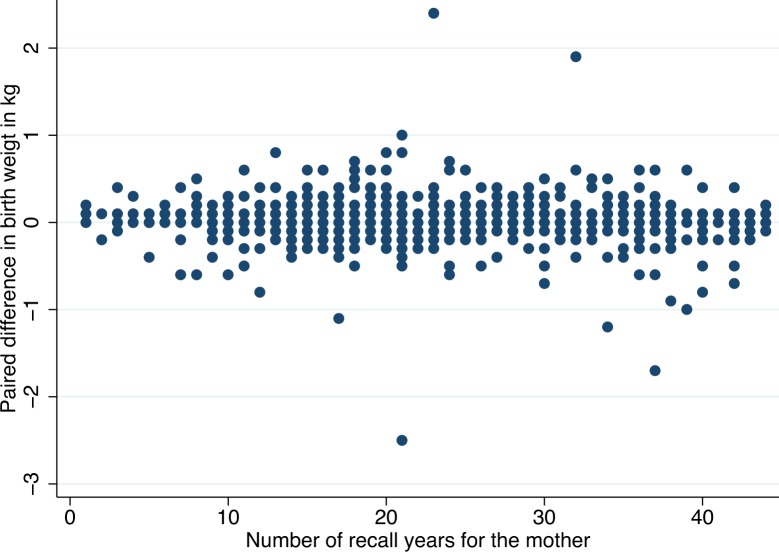
Paired difference between mothers reported birth weight and birth weight in the MBRN plotted against the number of recall years for the mother in 1464 children with valid data in both data sources.

The paired difference between reported and registry birthweight data did not change markedly with an increasing number of recall years (Fig 2), and the correlation coefficient between paired difference in birthweight and number of recall years was not significant (correlation = -0.05, p = 0.06). Apart from some obvious outliers the differences looked more or less randomly distributed (Fig 2).

**Fig 3:**
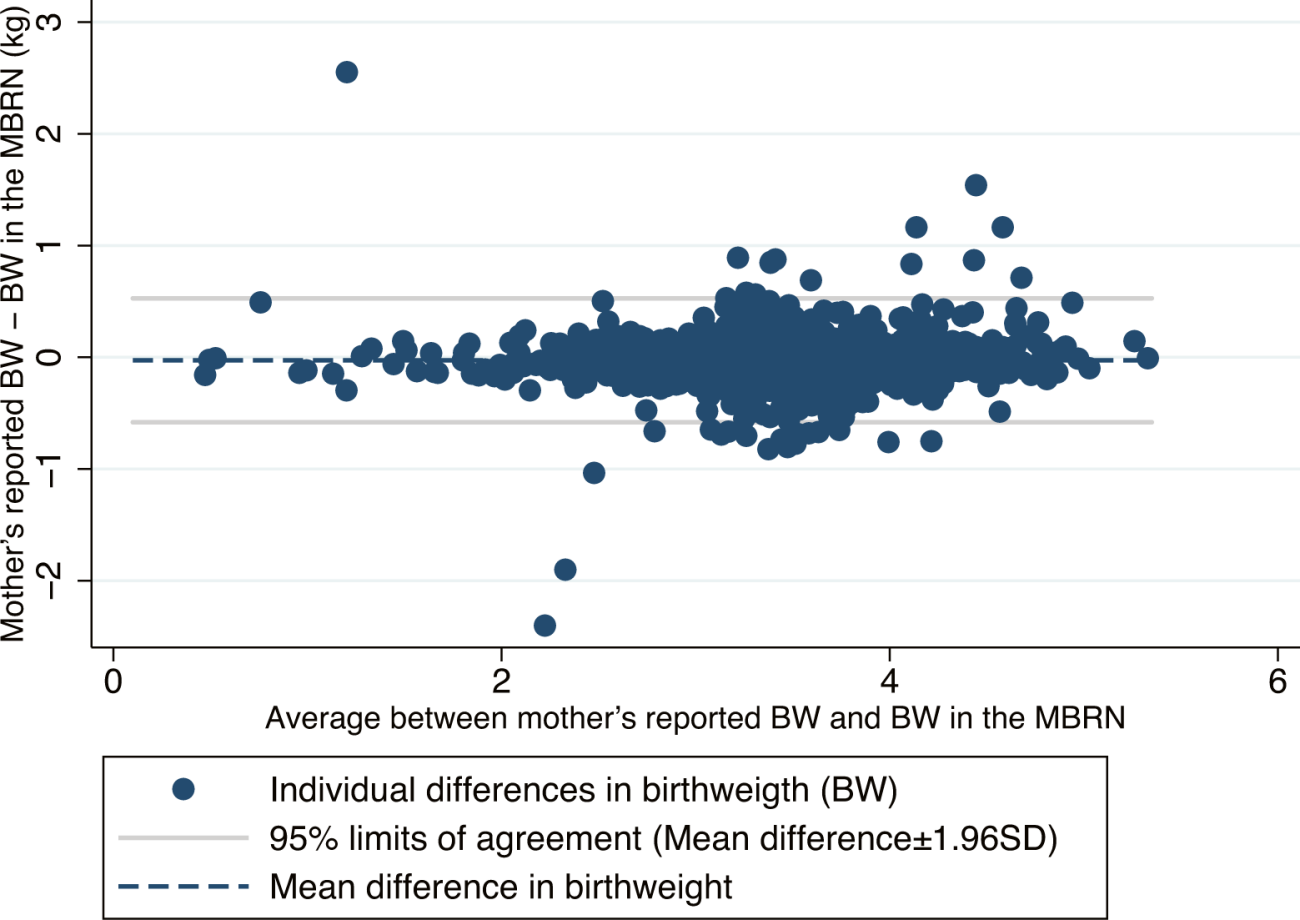
Bland-Altman plot for birth weight showing the paired difference between mothers reported birth weight and birth weight in the MBRN plotted against the average of the paired birth weights. The mean difference is plotted as a dashed line. Systematic bias is indicated by the distance between the dashed line and y = 0. Limits of agreement is calculated as mean difference ± 1.96 times the standard deviation of the paired differences, corrected for clustering within mother’s.

The Bland-Altman plot in Fig 3 shows the paired difference in birthweight (mother’s reported birthweight minus birthweight in the MBRN) against the mean of the paired birthweights. Apart from the same obvious outliers as in Fig 2, the differences were largest for children with birthweight in the range 3.0–4.5 kg, but there was no clear association between the size of the difference—the error in reporting—and the birthweight of the child.

For 81% of the children, mother’s recalled birthweight was within ± 0.1 kg from the birthweight recorded in the registry, and for 90% reported birthweight was within 0.2 kg of recorded birthweight (Table 4).

**Table 4:** Difference between mother’s recall of birth weight and birth weight in the MBRN in 1464 children with birth weight registered both in the questionnaire and the MBRN).

Difference from birthweight in MBRN	Maternal recall	
	n	%
0.0 kg	756	51.6
± 0.1 kg	1188	81.2
± 0.2 kg	1310	89.5
± 0.3 kg	1372	93.7
± 0.4 kg	1412	96.5
± 0.5 kg	1427	97.5

## Discussion

In this study of maternally reported birth and pregnancy characteristics in a population based questionnaire study as validated against the Medical Birth Registry of Norway, we found very good agreement for the important variables: Gender, birthweight, instrumental delivery and caesarean section. We also found good agreement for preterm birth, and moderate agreement for induction of labour. The agreement was poor for the reporting of postterm delivery. While the sensitivity varied, the specificity of all maternally recorded variables was very high. The recall of birthweight was independent of time since delivery, even for deliveries almost fifty years ago. Validity of maternally recorded low birthweight and pre-term birth did not differ by the child’s asthma status, and risk associations of low birthweight and preterm birth with asthma in the child were similar for maternally recorded and registry recorded data.

Birthweight is an important parameter in studies of early life events and impact on future health and disease. We found a very good agreement in reported birthweight, with 81% of recalled birthweight within ± 100 g compared to registry data, and 90% within ± 200 g. In a Danish study, Adegboye and Heitmann also reported good agreement concerning birthweight in a group of children aged 8–18 years (n = 1428) [19]. They found that 92% of recalled birthweights were within ± 100 g compared to data from the Danish Birth Registry. Overbeek et al. found a high intra-class correlation coefficient for birthweight in a Dutch report on validity of maternal data on pregnancies for childhood cancer survivors [3]; the self-reported birthweights tended to be higher than data reported in the Netherlands Perinatal Registry (PRN). In a British survey of children aged 4–9 years, substantial agreement between maternal report in postal questionnaires and information obtained from antenatal records was found regarding birthweight (kappa 0. 986) [4]. In a recent study from New South Wales, Australia, a 98% agreement (kappa 0.73) was found for children with low birthweight (<2500 g), comparing self-reported survey data with the Perinatal Data Collection [20]. It is notable that the mothers’ recall of their own birthweight is not similarly good [21].

Preterm birth is another variable of considerable interest. In our study the agreement for births before 32 weeks of gestation was better than that for births before week 37. Birth before week 32 implies that the newborn has to stay for a period in the neonatal intensive care unit, which is likely to be remembered by the parents. Extreme prematurity has the heaviest impact on future health, and thus, correct reports about this variable is important for research on this vulnerable group. The above mentioned study from Australia showed a 90% agreement (kappa 0.52) for live birth with gestational age less than 37 weeks, which is lower than in our study [20]. A French study including 530 mothers, showed an agreement with kappa 0.85 when comparing maternal reports to medical reports for preterm birth <32 weeks of gestation [9]. In this study data collection was done as early as 6 weeks after delivery, which may explain the better results compared to our study (kappa 0.73) where the mean [1, 2] number of years since delivery was 22 years at the time of data collection.

There is currently a considerable interest in postterm delivery related to the discussion of the optimal timing of inducing labour. Ours is one of the few studies investigating the validity of recalled postterm delivery. Unfortunately, our analyses showed poor agreement between maternal recall and MBRN data for this parameter. Of 237 deliveries reported to be postterm, only 103 (43%) occurred at 42 weeks of gestation or later, thus, according to the MBRN the remaining 134 were misclassified. Eight of the deliveries reported as postterm took place before 40 weeks (week 37–39) while the majority took place in week 40 (n = 36, 27%), and in week 41 (n = 90, 67%). Thus, most of the misclassified births reported to be postterm, took place after the term date, but not from week 42 or later as defined by the WHO as postterm. In our study population there appears to be a prevalent misconception regarding the definition of postterm birth. Our findings are in contradiction to findings in a Danish study showing that 85% of the morthers correctly reported postterm pregnancies [19].

For 85 of 1714 children reported by the mothers, data were not found in the birth registry. There are several possible reasons for non-match: The mothers were asked to include stillbirths and may have included spontaneous abortions before week 16 which are not included in the MBRN. Children born outside of Norway would not be included in the MBRN. The mother could have reported the wrong birth year or her handwriting could have been misinterpreted by the technician who transferred the data from the questionnaires to the database. It is unlikely that non-match would be of notable importance for the results on validity and risk-associations in our study.

In conclusion, maternally reported pregnancy and birth characteristics seem to be valid, even if the birth took place many years back in time and independent of the child’s disease status (exemplified with the child’s asthma status). The only exception was the reporting of postterm birth which was poor, mostly likely due to the discrepancy between the medical definition and the perception in the general public in this study population. Assessing birth and pregnancy data information from maternal reports, which is a feasible and cheap method, appears to be a useful method of high validity for several important birth and pregnancy parameters.

## Supporting information

S1 Table Validity of dichotomous pregnancy and birth- characteristics reported by the mothers in RHINE III stratified by recall years, with information in the MBRN used as the gold standard. (DOCX)

## References

[1] Barker DJ, Godfrey KM, Fall C, Osmond C, Winter PD, Shaheen SO. Relation of birth weight and childhood respiratory infection to adult lung function and death from chronic obstructive airways disease. BMJ. 1991;303(6804):671–5. ; PubMed Central PMCID: PMCPMC1670943. 1912913 10.1136/bmj.303.6804.671 PMC1670943

[2] Forsdahl A. Are poor living conditions in childhood and adolescence an important risk factor for arteriosclerotic heart disease? Br J Prev Soc Med. 1977;31(2):91–5. ; PubMed Central PMCID: PMCPMC479002. 884401 10.1136/jech.31.2.91 PMC479002

[3] Overbeek A, van den Berg MH, Hukkelhoven CW, Kremer LC, van den Heuvel-Eibrink MM, Tissing WJ, et al Validity of self-reported data on pregnancies for childhood cancer survivors: a comparison with data from a nationwide population-based registry. Hum Reprod. 2013;28(3):819–27. doi: 10.1093/humrep/des405 . 23175500 10.1093/humrep/des405

[4] Rice F, Lewis A, Harold G, van den Bree M, Boivin J, Hay DF, et al Agreement between maternal report and antenatal records for a range of pre and peri-natal factors: the influence of maternal and child characteristics. Early Hum Dev. 2007;83(8):497–504. doi: 10.1016/j.earlhumdev.2006.09.015 . 17071023 10.1016/j.earlhumdev.2006.09.015

[5] Olson JE, Shu XO, Ross JA, Pendergrass T, Robison LL. Medical record validation of maternally reported birth characteristics and pregnancy-related events: a report from the Children's Cancer Group. Am J Epidemiol. 1997;145(1):58–67. . 8982023 10.1093/oxfordjournals.aje.a009032

[6] Quigley MA, Hockley C, Davidson LL. Agreement between hospital records and maternal recall of mode of delivery: evidence from 12 391 deliveries in the UK Millennium Cohort Study. BJOG. 2007;114(2):195–200. doi: 10.1111/j.1471-0528.2006.01203.x . 17166217 10.1111/j.1471-0528.2006.01203.x

[7] O'Sullivan JJ, Pearce MS, Parker L. Parental recall of birth weight: how accurate is it? Arch Dis Child. 2000;82(3):202–3. PubMed Central PMCID: PMCPMC1718239. doi: 10.1136/adc.82.3.202 10685920 10.1136/adc.82.3.202 PMC1718239

[8] Kristensen P, Irgens LM. Maternal reproductive history: a registry based comparison of previous pregnancy data derived from maternal recall and data obtained during the actual pregnancy. Acta Obstet Gynecol Scand. 2000;79(6):471–7. . 10857871

[9] Troude P, L'Helias LF, Raison-Boulley AM, Castel C, Pichon C, Bouyer J, et al Perinatal factors reported by mothers: do they agree with medical records? Eur J Epidemiol. 2008;23(8):557–64. doi: 10.1007/s10654-008-9268-9 . 18560979 10.1007/s10654-008-9268-9

[10] Svanes C, Dharmage S, Sunyer J, Zock JP, Norback D, Wjst M, et al Long-term reliability in reporting of childhood pets by adults interviewed twice, 9 years apart. Results from the European Community Respiratory Health Survey I and II. Indoor Air. 2008;18(2):84–92. doi: 10.1111/j.1600-0668.2008.00523.x . 18333988 10.1111/j.1600-0668.2008.00523.x

[11] Johannessen A, Verlato G, Benediktsdottir B, Forsberg B, Franklin K, Gislason T, et al Longterm follow-up in European respiratory health studies—patterns and implications. BMC Pulm Med. 2014;14:63 doi: 10.1186/1471-2466-14-63 ; PubMed Central PMCID: PMCPMC4021078. 24739530 10.1186/1471-2466-14-63 PMC4021078

[12] WHO. International statistical classification of diseases and related health problems, tennth revision (ICD 10) Geneva: World Health Organization, 1992.

[13] Thomsen LC, Klungsoyr K, Roten LT, Tappert C, Araya E, Baerheim G, et al Validity of the diagnosis of pre-eclampsia in the Medical Birth Registry of Norway. Acta Obstet Gynecol Scand. 2013;92(8):943–50. doi: 10.1111/aogs.12159 . 23621424 10.1111/aogs.12159

[14] Baghestan E, Bordahl PE, Rasmussen SA, Sande AK, Lyslo I, Solvang I. A validation of the diagnosis of obstetric sphincter tears in two Norwegian databases, the Medical Birth Registry and the Patient Administration System. Acta Obstet Gynecol Scand. 2007;86(2):205–9. doi: 10.1080/00016340601111364 . 17364284 10.1080/00016340601111364

[15] Engeland A, Bjorge T, Daltveit AK, Vollset SE, Furu K. Validation of disease registration in pregnant women in the Medical Birth Registry of Norway. Acta Obstet Gynecol Scand. 2009;88(10):1083–9. doi: 10.1080/00016340903128454 . 19657758 10.1080/00016340903128454

[16] Vikanes A, Magnus P, Vangen S, Lomsdal S, Grjibovski AM. Hyperemesis gravidarum in the Medical Birth Registry of Norway—a validity study. BMC Pregnancy Childbirth. 2012;12:115 doi: 10.1186/1471-2393-12-115 ; PubMed Central PMCID: PMCPMC3534526. 23095718 10.1186/1471-2393-12-115 PMC3534526

[17] Klungsoyr K, Harmon QE, Skard LB, Simonsen I, Austvoll ET, Alsaker ER, et al Validity of pre-eclampsia registration in the medical birth registry of norway for women participating in the norwegian mother and child cohort study, 1999–2010. Paediatr Perinat Epidemiol. 2014;28(5):362–71. doi: 10.1111/ppe.12138 ; PubMed Central PMCID: PMCPMC4167249. 25040774 10.1111/ppe.12138 PMC4167249

[18] Bland JM, Altman DG. Agreement between methods of measurement with multiple observations per individual. J Biopharm Stat. 2007;17(4):571–82. doi: 10.1080/10543400701329422 . 17613642 10.1080/10543400701329422

[19] Adegboye AR, Heitmann B. Accuracy and correlates of maternal recall of birthweight and gestational age. BJOG. 2008;115(7):886–93. doi: 10.1111/j.1471-0528.2008.01717.x ; PubMed Central PMCID: PMCPMC2438372. 18485168 10.1111/j.1471-0528.2008.01717.x PMC2438372

[20] Gresham E, Forder P, Chojenta CL, Byles JE, Loxton DJ, Hure AJ. Agreement between self-reported perinatal outcomes and administrative data in New South Wales, Australia. BMC Pregnancy Childbirth. 2015;15:161 doi: 10.1186/s12884-015-0597-x ; PubMed Central PMCID: PMCPMC4524430. 26238999 10.1186/s12884-015-0597-x PMC4524430

[21] Tehranifar P, Liao Y, Flom JD, Terry MB. Validity of self-reported birth weight by adult women: sociodemographic influences and implications for life-course studies. Am J Epidemiol. 2009;170(7):910–7. doi: 10.1093/aje/kwp205 ; PubMed Central PMCID: PMCPMC2765356. 19748903 10.1093/aje/kwp205 PMC2765356

