# Supplementary material for: Validation of maternal reported pregnancy and birth characteristics against the Medical Birth Registry of Norway
Source: PLoS One. 2017 Aug 4;12(8):e0181794. doi: 10.1371/journal.pone.0181794 (PMC5544234; doi:10.1371/journal.pone.0181794)
Supplement: S1 Table — (DOCX) [file pone.0181794.s001.docx]

**S1 Table**. **Validity of dichotomous pregnancy and birth- characteristics reported by the mothers in RHINE III stratified by recall years, with information in the MBRN used as the gold standard**

|  |  | Prevalence in questionnaire | Sensitivity (95% CI) | Specificity (95% CI) | PPV (95% CI) | NPV (95% CI) | Kappa |
| --- | --- | --- | --- | --- | --- | --- | --- |
| Recall≤ 20 years | |  |  |  |  |  |  |
|  | Boys | 339/679 | 99.7 (98.4-100) | 99.7 (98.4-100) | 99.7 (98.4-100) | 99.7 (98.4-100) | 0.99 |
|  | Preterm delivery | 50/653 | 78.1 (60.0-90.7) | 96.0 (94.1-97.4) | 50 (35.5-64.5) | 98.8 (97.6-99.5) | 0.59 |
|  | Extremely preterm delivery (<32 weeks) | 3/653 | 60.0 (14.7-94.7) | 100 (99.4-100) | 100 (29.2-100) | 99.7 (99.9-100) | 0.75 |
|  | Postterm delivery (>42 weeks) | 89/653 | 55.0 (41.6-67.9) | 90.6 (87.9-92.8) | 37.1 (27.1-48.0) | 95.2 (93.1-96.8) | 0.37 |
|  | Induction of labour | 131/690 | 78.3 (65.8-87.9) | 86.7 (83.8-89.2) | 35.9 (27.7-44.7) | 97.7 (96.1-98.8) | 0.42 |
|  | Use of forceps | 28/651 | 83.3 (62.6-95.3) | 98.7 (97.5-99.4) | 71.4 (51.3-86.8) | 99.4 (98.4-99.8) | 0.76 |
|  | Use of vacuum | 20/651 | 70.4 (49.8-86.2) | 99.8 (99.1-100.0) | 95.0 (75.1-99.9) | 98.7(97.5-99.5) | 0.80 |
|  | Caesarean section | 89/691 | 100 (95.8-100) | 99.6 (98.7-100) | 97.8 (92.1-99.7) | 100 (99.3-100) | 0.99 |
| Recall> 20 years | |  |  |  |  |  |  |
|  | Boys | 412/885 | 99.4 (98.2-99.9) | 99.5 (98-3-99.9) | 99.6 (98.5-99.9) | 99.3 (97.9-99.8) | 0.99 |
|  | Preterm delivery | 72/863 | 70.2 (56.6-81.6) | 96.0 (94.4-97.3) | 55.6 (43.4-67.3) | 97.9 (96.6-98.7) | 0.59 |
|  | Extremely preterm delivery (<32 weeks) | 22/863 | 78.9 (54.4-93.9) | 99.2 (98.3- 99.7) | 68.2 (45.1 - 86.1) | 99.5 (98.8-99.9) | 0.73 |
|  | Postterm delivery (>42 weeks) | 148/863 | 48.3 (39.9-56.7) | 89.1 (86.6 – 91.3) | 47.3 (39.0-55.7) | 89.5 (87.0- 91.7) | 0.37 |
|  | Induction of labour | 181/930 | 66.2 (57.3-74.2) | 88.1 (85.7 – 90.3) | 47.5 (40.1-55.1) | 94.1 (92.2-95.7) | 0.47 |
|  | Use of forceps | 55/881 | 82.0 (68.6-91.4) | 98.3 (97.2-99.1) | 74.5 (61.0- 85.3) | 98.9 (97.9-99.5) | 0.77 |
|  | Use of vacuum | 17/881 | 83.3 (58.6-96.4) | 99.8 (99.2-100.0) | 88.2 (63.6-98.5) | 99.7 (99.0-99.9) | 0.85 |
|  | Caesarean section | 63/881 | 100 (94.3-100) | 100 (99.6-100) | 100 (94.3-100) | 100 (99.6-100) | 1.00 |
